# Supplementary material for: Education and income-related inequalities in multimorbidity among older Brazilian adults
Source: PLoS One. 2022 Oct 13;17(10):e0275985. doi: 10.1371/journal.pone.0275985 (PMC9560520; doi:10.1371/journal.pone.0275985)
Supplement: Attachment — Submitted filename: Response to Reviewers.doc [file pone.0275985.s001.doc]

**Journal Requirements:**

*Please ensure that your manuscript meets PLOS ONE's style requirements, including those for file naming. The PLOS ONE style templates can be found at 
https://journals.plos.org/plosone/s/file?id=wjVg/PLOSOne_formatting_sample_main_body.pdf and* 
[*https://journals.plos.org/plosone/s/file?id=ba62/PLOSOne_formatting_sample_title_authors_affiliations.pdf*](https://journals.plos.org/plosone/s/file?id=ba62/PLOSOne_formatting_sample_title_authors_affiliations.pdf)*.*

**Response:** We have formatted the manuscript according to the journal’s style

*2. Please provide additional details regarding participant consent. In the ethics statement in the Methods and online submission information, please ensure that you have specified what type you obtained (for instance, written or verbal, and if verbal, how it was documented and witnessed). If your study included minors, state whether you obtained consent from parents or guardians. If the need for consent was waived by the ethics committee, please include this information.*

**Response:** The information is included in the ethics statement in the Methods.

*3. Thank you for stating the following financial disclosure: 
FBA - This work was supported by Foundation for Research Support from Minas Gerais State (FAPEMIG), Brazil [grant APQ-00573-21]. 
http://www.fapemig.br/pt/
Funder role: none.*

*Please state what role the funders took in the study.  If the funders had no role, please state: "**The funders had no role in study design, data collection and analysis, decision to publish, or preparation of the manuscript." 
If this statement is not correct you must amend it as needed. 
Please include this amended Role of Funder statement in your cover letter; we will change the online submission form on your behalf.*

**Response:** We have included the funder’s role in the cover letter

*4. Please review your reference list to ensure that it is complete and correct. If you have cited papers that have been retracted, please include the rationale for doing so in the manuscript text, or remove these references and replace them with relevant current references. Any changes to the reference list should be mentioned in the rebuttal letter that accompanies your revised manuscript. If you need to cite a retracted article, indicate the article’s retracted status in the References list and also include a citation and full reference for the retraction notice.*

**Response:** We have reviewed the reference list.

**Reviewer #1:**

*Bof de Andrade and colleagues conducted a cross-sectional analysis to explore the association between socioeconomic inequalities and the prevalence of multimorbidity in the general population aged 60 years or older, using nationally-representative data retrieved from the most recent Brazilian National Health Survey. The study findings suggest that individuals with higher socioeconomic status tend to have a lower prevalence of multimorbidity in Brazil.*

*The study carries important implications for health equity across different regions, despite room for improvement through a minor revision.*

*The estimation of the slope index of inequality (SII) and the concentration index (CI), which were adopted as measures of inequality in the study, were not explicitly explained. For example, it remains to be elucidated as to how the regression models were constructed to yield the inequality coefficient. Besides, the authors may wish to add a bit more elaboration on how the confounding factors were accounted for in the study.*

**Response:** Thanks for the careful review of our manuscript and for the suggestions. We have included more information on how to estimate the indexes and included references for the papers that detailed explained the methods. Regarding the confounders, they were not discussed because in these analyses only the outcome and the socioeconomic position measures are included.

**Reviewer #2:**

*The lines in the article are not numbered.*

**Response:** Thanks for the careful review of our manuscript and for the suggestions. We have included the lines numbers in the paper.

*In the abstract the conclusion should provide more information on the distribution of multimorbidity in the country, as it is, the conclusion is to general and more detailed information on the most affected areas of the country could be helpful.*

**Response:** Thanks for the suggestion. We included this information in the abstract.

*In the first paragraph of the introduction reference 4 is in () and not in [].*

**Response:** We have made the change.

*Methods: In the independent variables, the education variable is defined with six categories, but one category is missing in the parenthesis, but then it is mentioned in table 1. Add the category in the text “**some college or more”.*

**Response:** We have included the category.

*In the discussion, a closing paragraph should be included regarding the inequality differences found in the different regions of the country and its relationship with the high multimorbidity prevalence. This was not evident in the discussion.*

**Response:** We highlighted this information in the discussion section in the paragraph where the magnitude of the inequalities is discussed.

*There is no conclusion in the manuscript, include it*

**Response:** We have reviewed the discussion to better address the conclusions.
